# Predicting age and gender from network telemetry: Implications for privacy and impact on policy

**DOI:** 10.1371/journal.pone.0271714

**Published:** 2022-07-21

**Authors:** Lida Kuang, Samruda Pobbathi, Yuri Mansury, Matthew A. Shapiro, Vijay K. Gurbani

**Affiliations:** 1 Department of Computer Science, Illinois Institute of Technology, Chicago, IL, United States of America; 2 Department of Social Sciences, Illinois Institute of Technology, Chicago, IL, United States of America; Ardakan University, IRAN, ISLAMIC REPUBLIC OF

## Abstract

The systematic monitoring of private communications through the use of information technology pervades the digital age. One result of this is the potential availability of vast amount of data tracking the characteristics of mobile network users. Such data is becoming increasingly accessible for commercial use, while the accessibility of such data raises questions about the degree to which personal information can be protected. Existing regulations may require the removal of personally-identifiable information (PII) from datasets before they can be processed, but research now suggests that powerful machine learning classification methods are capable of targeting individuals for personalized marketing purposes, even in the absence of PII. This study aims to demonstrate how machine learning methods can be deployed to extract demographic characteristics. Specifically, we investigate whether key demographics—gender and age—of mobile users can be accurately identified by third parties using deep learning techniques based solely on observations of the user’s interactions within the network. Using an anonymized dataset from a Latin American country, we show the relative ease by which PII in terms of the age and gender demographics can be inferred; specifically, our neural networks model generates an estimate for gender with an accuracy rate of 67%, outperforming decision tree, random forest, and gradient boosting models by a significant margin. Neural networks achieve an even higher accuracy rate of 78% in predicting the subscriber age. These results suggest the need for a more robust regulatory framework governing the collection of personal data to safeguard users from predatory practices motivated by fraudulent intentions, prejudices, or consumer manipulation. We discuss in particular how advances in machine learning have chiseled away a number of General Data Protection Regulation (GDPR) articles designed to protect consumers from the imminent threat of privacy violations.

## 1 Introduction and motivation

Users of social media, cell phones, home security cameras, location trackers, among other devices are likely not aware of the implications of having massive amounts of personal information compiled by service providers. They are definitely unaware that this compiled data can be recreated by third parties through the application of machine learning (ML) techniques, a disciplinary area in artificial intelligence (AI). The implication is that people—most of whom value their online privacy [1], albeit in varying degrees [2]—are constantly at risk from having even their anonymized information de-anonymized by ML techniques. As we critically examine the principles of a regulatory environment to safeguard privacy, the following overarching questions are considered in this paper: *How easily can these techniques be applied, and what can be done at the policy level to thwart such efforts?*

Telecommunications is increasingly conducted using Internet Protocol (IP) technologies, the same protocols and networks that power the Internet. With the proliferation of mobile phones globally, many commercial companies and governments seek to collect and analyze the metadata generated by users (or subscribers) who interact with the network. While service providers (i.e., companies that provide the mobile phones as well as access to the telecommunications network) may have demographic information on their users, governments and other corporations do not. (In some cases, even service providers do not have access to the demographic information of those using their network, e.g., pre-paid SIM cards are not always registered to a user (the U.S. case.) Predicting user demographics, especially in terms of age and gender, remains an attractive outcome, as service providers can use it to tailor marketing material that would then be deployed to convert pre-paid subscribers to post-paid (monthly) subscribers. Of course, demographics can also be used for targeted advertising.

Our examination begins with a focus on the potential for ML techniques to identify key attributes of individuals from an anonymized dataset of cell phone users in a Latin American country. We account for attributes of social data, “an umbrella concept for all kinds of digital traces produced by or about users, with an emphasis on content explicitly written with the intent of communicating or interacting with others” [3]. It has long been known that we can make all sorts of predictions about users from their cell phone data, namely the user’s geographic location [4], and we do not dispute the fact that there may be times when it is useful, even life-saving, to have access to users’ personal information [4]. (For example, access to these data has been particularly important for automated contact tracing efforts during the COVID-19 pandemic in places like South Korea and Singapore [5, 6].) But if the firm has adverse motivations [7], or if a third-party is able to access data in some manner and make predictions about user demographics, the potential costs of identity or physical theft could be enormous. There also exists the possibility that telecommunications firms are incentivized to use this information for its marketing potential, selling it to other firms [8]. As an example, third-party trackers have been shown to much more effectively follow users on healthcare websites [9], implying that users may be targeted and tracked for tailored marketing campaigns.

We treat privacy-related matters about telemetry data with a posture of caution [10]. Reflecting the biases at work when one attempts to identify users via facial recognition technology [11–13], we acknowledge the potential for entities to use user-based information to infer additional attributes about users in order to discriminate against particular racial, ethnic, or gender groups. This is evident from cases involving access to credit, employment opportunities, and health care services in the United States [14]. (Even ostensibly inclusive advertisements highlighting employment and housing opportunities on Facebook have been susceptible to this bias [15] This may be even more pronounced with regard to clinical research and vulnerable populations [16–18].) There are also compounding and marginalizing effects on youth of color [19].

Of course, when an individual opts into a particular service, some of these conditions regarding basic privacy-related protections (i.e. non-ML-related concerns) are explicitly stated in the service agreement. Yet, most people check the “I agree” box without so much as giving the agreements even a casual reading [20, 21]. These service agreements thus ineffectively inform the users about what is at stake, particularly in terms of the kinds of privacy losses examined here [22]. In response, we are adamant that the government implement policies to counter private-sector privacy violations in line with the European Union’s General Data Protection Regulation (GDPR [23]). There has been resistance to these types of policies with some in the private sector actively lobbying against their implementation [8]. Events such as the Cambridge Analytica scandal [24], while not limited to cell phone-based network telemetry data, only highlight the need for government intervention and protections from predatory firms, particularly if analogous sets of data enable private parties to accurately predict individual voting behavior [25]. (*Telemetry* is the science of transmitting measurements from a source to a destination for storage and subsequent analysis. The use of telemetry in networks is not new, but its importance has grown in relation to the data volumes generated by user activities from various networked devices).

This does not preclude the possibility that such efforts at identifying users through ML techniques may be made by governments attempting to monitor the public, both overtly and covertly. In line with the censoring of information disseminated and shared online, reflected in China [26, 27], electronic surveillance by the government can be justified on the basis of national security [28]. There is evidence of this in the history of the Vietnam War [29] and, much later, by Edward Snowden [30], the latter of which has parallels to the present telephony-focused study through the U.S. National Security Agency’s use of Verizon’s metadata [31, 32]. To be explicit, governments have used this technology for control and repression [33, 34].

Our contributions cross several domains, ultimately building on the literature on machine learning techniques and privacy-related concerns.

1. Given the existence of ML-based techniques to de-anonymize data processing, and given the lack of policy-related attention to this issue, this paper motivates a larger conversation about corporations and governmental institutions that are assigned the task of protecting privacy-related violations but fail because they ignore the role of ML techniques.
2. We use the telecommunications domain as a case, employing the assumption that third-party adversaries rather than service providers are doing the modeling, to show the relative ease by which deep learning models can be used to predict age and gender of users using network telemetry data. Automatic gender recognition (“gender classification”) raises its own fairness-related concerns, particularly the use of visual and voice-based data (i.e., for transgender-identifying individuals) [35, 36].
3. The computational algorithms used here can diminish expectations of privacy disproportionately across subsets of the population. In response, we offer the following insights when prescribing policy: regulations must be designed especially to help prevent the targeting of younger or older individuals who are particularly susceptible to scams [37].
4. To date, governments in general have insufficiently addressed these privacy-related violations. Thus, our focus especially on oversight of the private sector’s management of user information as well as how to properly anticipate and respond to the privacy challenges associated with ML-based techniques in the future is a crucial update to existing research.

The rest of the paper is structured as follows: Section 2 places our effort in the context of ongoing work in privacy, machine learning, and regulations. Section 3 presents our dataset and the models for age and gender prediction. Section 4 examines the issues surrounding the use of machine learning techniques as a policy tool, discusses the promise and limits of GDPR, while Section 5 proposes policy prescriptions that may enable machine learning and GDPR to work in synergy for protecting user privacy followed by a discussion in Section 6. We conclude with closing thoughts in Section 7.

## 2 Literature review and related work

Efforts to predict demographic qualities of anonymous individuals through other artifacts of the individual have a long history, beginning with Juan Huarte de San Juan’s 1575 book on European graphology (handwriting analysis) [38], followed soon after by Camillo Baldi’s 1622 book focusing on the American case [39]. More recently, a study concluded in 1991 shows that there exists statistically relevant correlation between gender and handwriting style [40]. With the advent of the Internet and the availability of powerful computing resources and deep learning models, we are now able to use the digital traces of a user to predict personality characteristic and demographics. While some of this research focuses on predicting demographics from images [41, 42], patterns of walking (i.e. gait analysis [43]), or even keystroke and mouse movement analysis [44], we eschew topics like those in favor of research that utilizes aspects of mobile phones to predict user demographics.

We are also interested in social media-based functions that are typically associated with mobile phone use. For example, Krismayer et al. [45] investigate whether music listening habits of users of social music platform can be used to predict their age, gender, and nationality, and Shafiloo et al. [46] propose a framework to predict users’ demographics based on users’ movie ratings. Similarly, Wood-Doughty et al. [47] explore character-level neural models that learn a representation of a user’s name and screen name in Twitter to predict gender and ethnicity, while Culotta et al. [48] construct a dataset consisting of web traffic demographic data for the purpose of classifying individual Twitter users by ethnicity, gender, and political preference. Finally, Seneviratne et al. [49] collect a snapshot of installed applications on mobile phones and use the data to predict the gender of the user with 70% accuracy, while Malmi et al. [50], Sangaralingam et al. [51], and Qin et al. [52, 53] examine installed and regularly used mobile phone applications to predict age, race, gender, and income. In contrast, Hu et al. [54] predict the age and gender of mobile phone users based simply on their Internet browsing behaviour, while Wang et al. [55] predict gender by observing users’ mobile phone-based response times.

Coming at the issue from a slightly different approach, Akter and Holder [56] predict age (85.99%) and gender (66.45%) using GPS location data and examining those places visited by individuals. In such a graph, the nodes are the locations visited (bank, prison, fire station, etc.) and an edge between nodes indicates that the user visited both the nodes; yet, their dataset is comprised of only 185 observations and is dominated by males (61.78%) between the ages of 22 and 33 (almost 70% of the dataset). Combining a number of the approaches mentioned above, Ying et al. [57] use 45 behavioral features (average and maximum distance from home, kind and duration of music listed to per day) and environmental features (paired Bluetooth devices, wireless devices detected per day etc.) to predict gender and marital status.

The extant literature also uses back-end processes prevalent in telecommunications for demographic predictions. Felbo et al. [58] use a convolutional neural network to predict the age and gender of mobile phone subscribers. They demonstrate an accuracy of 79.7% for gender and 63.1% for age binned across three groups, namely, 18–39, 40–49, and 50+, based on eight features from call detail records (CDR) provided by the service provider. (A CDR is a data record produced by a communications server that contains various attributes of a call, such as the phone numbers of the parties engaged in the call, the time a call was made, type of call (e.g., voice), SMS, etc.) Al-Zubai et al. [59] use CDRs and related information like billing and customer relationship management systems to predict age (65.5% accuracy) and gender (85.6% accuracy). For their method to be useful, the predictors are extracted from the operating support systems (OSS) and business support systems (BSS). (OSS and BSS separate the network operations aspect from the business aspect; for example, a service provider’s OSS will ensure that the network is functioning smoothly while the BSS engages in customer relationship management and billing).

Table 1 summarizes the surveyed literature in this section. It should be pointed out that the literature discussed above does not approach the problem of age and gender prediction using network telemetry data or similar measures of how an individual interacts with the mobile phone network. Another major distinction of our work is that, unlike the surveyed literature, we eschew techniques that might violate the privacy of the user irrespective of the predictive value offered by such techniques. That is, we assume the presence of an adversarial model, while the existing literature typically assumes that the service provider is engaging in modeling and has access to back end OSS/BSS systems and CDRs, or that the user has acquiesced to having his or her applications monitored. Our work, though, assumes that the actor doing the modeling is likely to be a third-party adversary that does not need access to the user’s device or back-end systems for reconnaissance but can simply access the network from which it gathers the telemetry data needed to create a demographic prediction model; i.e. there is no need to access the user’s mobile device, the applications installed on it, or the back-end systems.

**Table 1:** Summary of literature surveyed.

Year	Author(s)	Demographics predicted	Artifacts used for modeling
1991	James Hartley [40]	Gender	Handwriting
2007	Hu et al. [54]	Age, gender	Browsing behaviour
2012	Ying et al. [57]	Gender, and marital status	Behavioral- and environmental features
2014	Qin et al. [53]	Age, gender	Applications usage
2015	Seneviratne et al. [49]	Gender	Installed applications
2015	Wang et al. [55]	Gender	Application response time
2016	Culotta et al. [48]	Age, gender, ethnicity, education, income, parental status, political preference	Twitter feed
2016	Malmi et al. [50]	Age, race, income	Installed applications
2017	Felbo et al. [58]	Age, gender	CDR records, OSS, BSS
2017	Akter et al. [56]	Age, gender	GPS location
2017	Avar Pentel [44]	Age, gender	Keystroke analysis and mouse movement
2018	Qin et al. [52]	Age, gender	Applications usage
2018	Sangaralingam et al. [51]	Age, gender	Installed applications
2018	Wood-Doughty et al. [47]	Gender, ethnicity	Twitter feed
2019	Rafique et al. [41]	Age, gender	Images
2019	Fang et al. [42]	Age, gender	Images
2019	Hamme et al. [43]	Age, gender	Gait analysis
2019	Krismayer et al. [45]	Age, gender, nationality	Music
2019	Al-Zubai et al. [59]	Age, gender	CDR records, OSS, BSS
2021	Shafiloo et al. [46]	Age, gender	Movie interests

Finally, buoyed by the “Brussels Effect” [60], GDPR, with its focus on user privacy, is increasingly being viewed as an important omnibus legislation for user privacy even outside the EU borders, as multinational corporations find it beneficial to apply a uniform privacy standard across multiple regulatory environments [61]. Given its importance, a number of papers have used GDPR to evaluate developments at the intersection of law and technology. Notably, Kotsios et al. [62] examine the consequences of GDPR on social network research, Gurbani et al. [63] evaluate the privacy of users interacting with a key network protocol in the context of GDPR, and Truong et al. [64] discuss a GDPR-compliant personal data management platform leveraging the emerging blockchain and smart contract technologies. This paper examines how advances in machine learning have chiseled away at GDPR, and proposes policy prescriptions to alleviate some of the losses.

## 3 Models for age and gender prediction

The machine learning (ML) revolution highlights out-of-sample predictive power using algorithms that are more efficient and accurate than traditional statistical methods [65]. ML methods that are becoming standard across disciplines for addressing supervised learning problems include decision trees, gradient boosting models, random forests, support vector machines, and neural networks. Such methods are optimized to perform well in areas that have experienced explosive growth in large-scale data [66].

**Fig 1:**
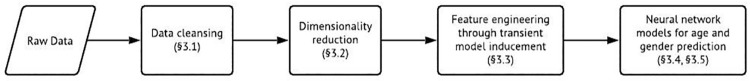
Summary of our approach to modeling.

Our proposed approach to modeling the age and gender prediction is summarized in Fig 1 and further described in details in the sections below. First, the raw data is obtained and rendered to be suitable for model induction as described in Section 3.1. Once cleansed, exploratory data analysis is performed to remove extraneous features and focus on those features of interest (Section 3.2). Next, we used ML algorithms such as XGBoost [67], LightGBM [68] and others to aid in feature engineering for age and gender as described in Section 3.3. Using the reduced number of features suggested by these algorithms, we then induce the final neural network models for age and gender, as described in Sections 3.4 and 3.5, respectively.

The exploratory data analysis and model building work described in the rest of this section was performed on a Linux (Fedora) operating system with 16 GB memory and an Intel i5–6200U CPUs with 4 cores. R version 3.6 [69] and Python version 2.7 [70] environments were used. The Keras Library release 2.2.5 with TensorFlow [71] was used for the deep neural network models.

### 3.1 Dataset description

Our dataset was provided by a wireless communications service provider from a population of pre-paid cellular phone users (or subscribers). It consisted of 14,549 observations with 1,686 dimensions, including the two response variables (age and gender). The response variables were obtained from customers surveys that solicited age and gender-related information. Each observation in the dataset contained the response variables and the predictor variables tracking the interactions between the user and the network. After cleaning the dataset, we were left with slightly more than 11,000 observations across 1,686 dimensions. The dataset was curated and given to us for modeling in this shape; we did not add any new dimensions. In fact, we reduced the dataset’s dimensionality as discussed in Sections 3.2 and 3.3.

The predictor variables are measures of how the subscriber interacted with the network, or *network telemetry*. These measures included, among others, subscriber interaction with applications like Whatsapp, Instagram, and Uber (in terms of bytes transferred over the network); subscriber interaction with banking applications (frequency); and subscriber interaction with sending and receiving SMS messages (frequency only, we were not provided any content of the SMS messages); and subscriber interaction with placing outbound phone calls and receiving inbound phone calls. Besides the network-related predictors that characterized the subscriber’s interaction with the network, some back office data was made available as predictors, e.g., how often the subscriber recharged (i.e. *topped up*, or purchased additional minutes on the pre-paid account), the average recharge amount, etc. The data was collected over a three month period during the late summer and early fall of 2017.

We characterize the 1,686 dimensions into *atomic* dimensions and *derivative* dimensions. Atomic dimensions exist independently and can be considered equivalent to the column rank of a matrix. On the other hand, derivative dimensions are derived dimensions, such as counts, percentages, and other linear combinations of the atomic dimensions.

To highlight how these atomic dimensions are extracted, consider the number of megabytes transferred over the network for the Uber ride-sharing application: This single atomic dimension generated 134 derivative dimensions, including moments of the atomic dimensions (e.g., average), aggregations of the atomic domain (e.g., Uber data as a percent of total data transferred, or percent of Uber data transmitted during the weekend versus weekdays), or derivative dimensions that are correlated with an atomic dimension (e.g., number of Uber sessions). Furthermore, to derive per-day derivative dimensions, a nychthemeral day (24 hours) is divided into four parts: P1 (one minute after midnight to 6:00 am), P2 (6:01 am to noon), P3 (12:01 pm to 6:00 pm), and P4 (6:01 pm to midnight), which gives rise to derivative dimensions such as the number of bytes used by the Uber application in P1, or P2, or P3, or P4, or even combinations such as P12, which is a summation of the number of bytes the Uber application used in P1 and P2. Similarly, other dimensions that measure network traffic related to applications such as Whatsapp or Instagram also generate 134 derivative dimensions per such application.

A portion of these 14,549 observations was incomplete, as some had null values, while others had missing values that had to be imputed. After removing those with missing values, we were left with approximately 11,000 observations. Given the number of dimensions (1,686), the curse of dimensionality is acute. (In machine learning, finding patterns from a finite number of samples in a high-dimensional feature space, with each feature having a range of possible values, requires a large training dataset to ensure that there are several samples with combination of values to learn from. The term *curse of dimensionality* itself was coined by Bellman when considering problems in dynamic programming [72].) To address the high dimensionality of a 1,686-variables dataset, we engaged in two phases of dimensionality reduction. In phase one, the reduction was made for exploratory data analysis, while in phase two, dimensionality reduction was performed for feature engineering purposes. These phases were independent of each other in that the second phase did not depend on the number of attributes resulting from the first phase. We detail in the following two subsections the procedures undertaken in each step.

### 3.2 Dimensionality reduction for exploratory data analysis

We focused first on the atomic dimensions through their correlations with the response variables. Specifically, we removed an atomic dimension containing personal identifying information (PII) such as a Mobile Station International Subscriber Directory Number, which do not explain variations in gender or age. (MSISDN). (MSISDN, or Mobile Station International Subscriber Directory Number is a unique number used to identify a mobile phone. This number includes a country code and a destination code within that country that identifies a subscriber. Since this information can identify a person, we removed it.) Having taken this step, we were left with 31 atomic dimensions. Finally, we dropped two atomic dimensions that did not correlate with either of the response variables, leaving us with 29 atomic predictors.

**Fig 2:**
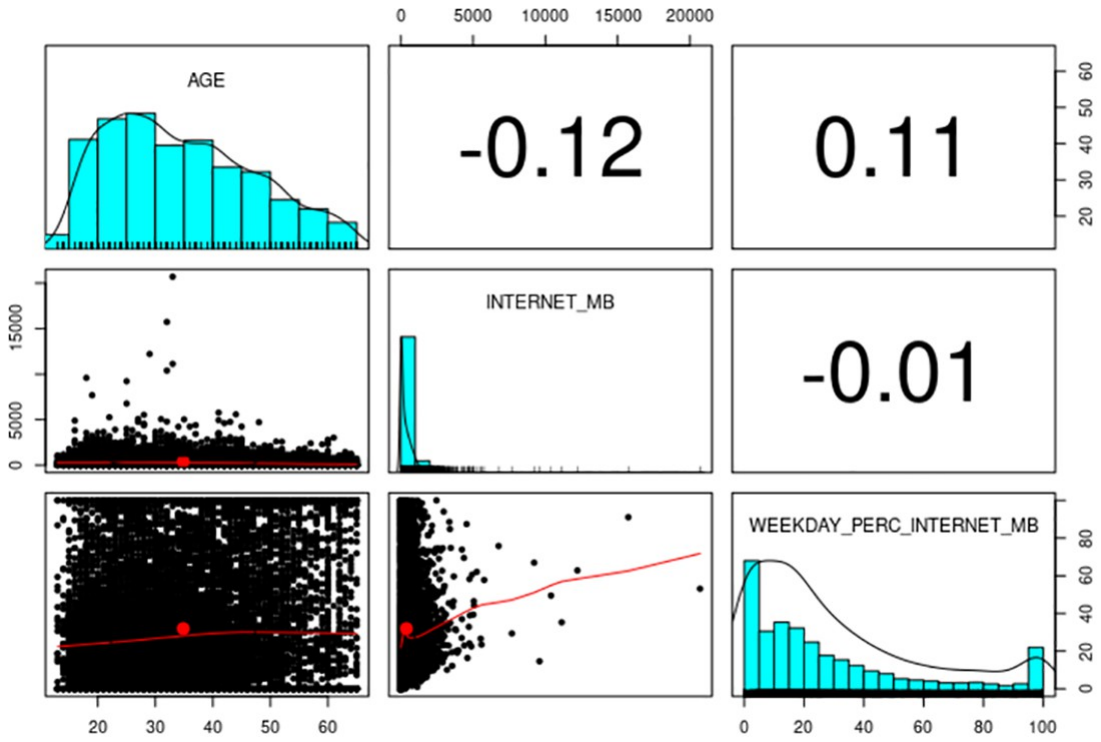
Correlation between response variable and atomic/derived dimensions.

Table 2 summarizes the 29 atomic dimensions that correlate with the response variables. Correlations with gender are performed using biserial correlations where Male = 1 and Female = 0. As the table shows, the correlations are not very strong overall; nonetheless, correlations between the 29 atomic dimensions and age are stronger than those between the former and gender. Note that derived attributes obtained from a linear combination of the atomic dimensions will not improve the response variables’ prediction and were thus discarded. Derived attributes that exhibit a quadratic relationship with the atomic dimensions marginally increased their correlations with the response variables and were therefore included in the list of predictors. More importantly, several derived attributes turned out to be correlated with the response variable independently of the correlations with the original atomic dimensions. For example, Fig 2 displays the correlations between age (response variable), Internet usage (in MB, an atomic variable), and the percentage of the Internet used on weekdays (a derived attribute). As the figure shows, age and general Internet usage are negatively correlated, but age and the derived weekdays’ usage are positively correlated.

**Table 2:** Response variable correlations. (For gender, Male = 1, Female = 0).

	Corr. with age	Corr. with gender
Payment method	-0.08	0.02
Number of devices	-0.06	0.04
Number of services	-0.23	0.00
Device type	0.03	-0.02
Device capability	-0.10	-0.02
Device age	-0.02	-0.03
Customer service	0.05	0.03
Need customer service	0.04	0.02
Recharge way	0.01	0.02
Main recharge way	0.00	0.02
Recharge value	0.00	0.06
Recharges	-0.06	0.01
Recharges Average	0.11	0.07
Social network (MB)	-0.18	0.05
Social network (number of sessions)	-0.19	0.03
Instagram (MB)	-0.07	0.00
Instagram (number of sessions)	-0.17	0.02
Whatsapp (MB)	-0.15	0.04
Whatsapp (number of sessions)	-0.17	0.03
Bank (MB)	-0.01	0.03
Bank (number of sessions)	-0.15	0.03
Uber (MB)	-0.02	0.03
Uber (number of sessions)	-0.15	0.03
Internet (MB)	-0.13	0.07
Internet (number of sessions)	-0.13	0.03
Received calls	0.06	0.07
Rec’d. call duration	0.05	0.00
Made calls	-0.01	0.01
Made calls duration	0.00	-0.15

**Fig 3:**
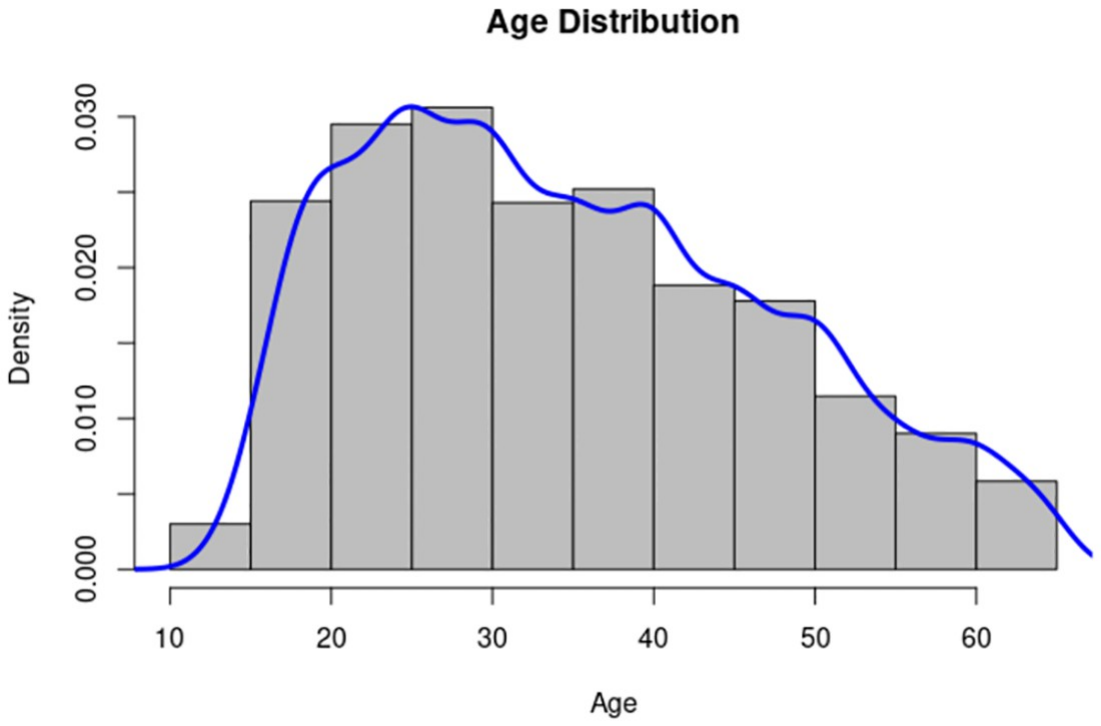
Age distribution in the dataset.

Finally, Fig 3 shows the age distribution in our dataset; from the figure, it is apparent that the dataset is skewed toward younger ages. The minimum age value among respondents in the dataset is 13 years (15 observations), and the maximum age in the dataset is 65 (64 observations). The binary distribution represented by gender is almost equally balanced between males and females.

### 3.3 Dimensionality reduction for feature engineering

**Fig 4:**
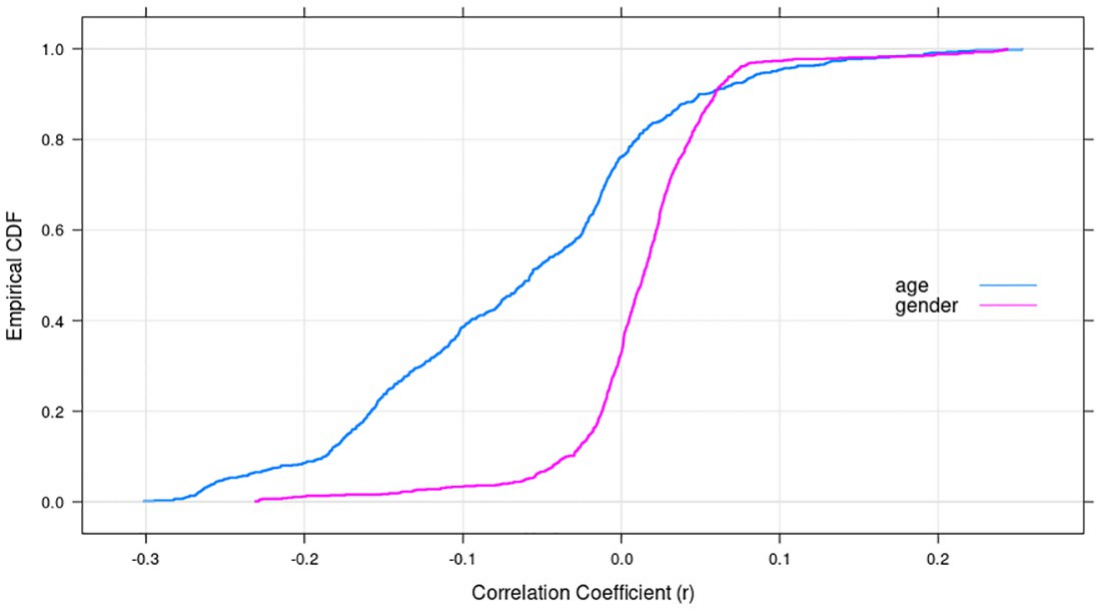
CDF for correlation of 749 attributes with response variables.

We identify next the variables to be included in the models through feature engineering. First, we removed the 293 features that had zero variance. We then examined the correlation coefficient for every pair of the remaining features, and randomly dropped one of the variables from each pair with a correlation coefficient of 0.98 or above. Doing so decreased the number of attributes to 749. We then computed the correlation of the response variables with these 749 variables and found the overall correlations to be weak. Table 3 presents the correlations between, respectively, the top-five most positively and negatively correlated attributes with the response variables, and Fig 4 shows the CDF of the correlated values of the 749 predictors with each response variable. Together, Tables 2 and 3, and Fig 4 show that the correlation between the response variables and the predictors is fairly weak; 78% of the predictors have a correlation between -0.30 and 0.0 with the response variable age, while 62% of the predictors have a correlation coefficient between 0.0 and 0.2 with the response variable gender.

**Table 3:** Correlation of surviving 749 features with the response variables.

	Positive correlation (Top 5)	Negative correlation (Top 5)	Avg.	Std. Dev
Gender	0.244, 0.242, 0.241, 0.238, 0.235	-0.231, -0.229, -0.229, -0.228, -0.227	0.122	0.054
Age	0.253, 0.224, 0.222, 0.220, 0.213	-0.301, -0.295, -0.283, -0.282, -0.282	-0.066	0.101

Next, we used LightGBM [68], a gradient boosting method library, to build a quick age prediction model primarily to identify the important features (gender was excluded as a feature here). The analysis of the model indicated that 704 attributes were detected as zero-importance features, and 32 were detected as low importance features. The remaining 11 explanatory attributes were used to develop the model for age prediction, including 10 that are derived dimensions and 1 that is an atomic dimension as discussed in Section 3.1. Table 4 presents the correlation coefficient for each of these features with age (in the table, P1-P4 refer to the division of nychthemeral time period into four parts as explained in 3.1).

**Table 4:** Correlation of age with features used for modeling.

	Dimension Type	Corr. Coefficient
Recharge Value	Atomic	0.06
Min Recharge Value	Derived	0.13
Calls made during P2	Derived	0.06
Percentage of days social network over-the-top applications used during P2	Derived	-0.29
Percentage of social network sessions used during P1	Derived	-0.07
Percent of received calls duration during P2	Derived	0.19
Percent of received calls duration during P4	Derived	-0.16
Percent of days where over-the-top calls were placed during P2 and P3	Derived	0.18
Percent of Internet megabytes used during the weekdays	Derived	0.11
Percentage of social network sessions used on weekends and holidays	Derived	-0.26
Whatsapp traffic (in MB) generated on weekends and holidays	Derived	-0.15

For gender modeling we followed the same procedure to remove 293 zero-variance features. Based on analysis of the correlation coefficients, we randomly selected one variable and dropped the other from each pair that exhibits a correlation of 0.95 and above. We then used the method of LightGBM [68] to analyze the features in a gender classification model, which allows us to settle on 111 features (age was not included in the feature list). Of these 111 features, five were atomic, while the remaining 96 were derived features. It is interesting to note that gender prediction required an order of magnitude more features than age prediction, explained in part by the low correlations that gender has with the atomic dimensions (as shown in Table 2).

### 3.4 Age prediction

We initially treated age prediction as a multivariate regression problem. Simple linear regression and quadratic regression techniques, however, generated predictions with high error measures and low accuracy. We therefore binned observations into 10 age categories, which allows us to frame the age modeling exercise as a classification problem. Subsequent application of a multinomial classification model to fit the data resulted in an accuracy of 18.43%, which was further improved to 25.93% using the limited memory Broyden-Fletcher-Goldfarb-Shanno optimization algorithm [73]. The use of different classifiers—specifically Random Forests [74], Support Vector Machines [75], multinomial logistic regression [76], and XGBoost [67]—did not improve model accuracy, which we set at the minimum threshold of 70% or above.

Since traditional classifiers did not meet the minimum threshold requirement, we resorted to artificial neural networks [77] to improve prediction accuracy. Neural networks, especially deep neural networks, are the state of art technique in use today for image classification [78], natural language processing [79], and privacy and security [80, 81]. Briefly, a neural network is a computing system consisting of a number of simple but highly connected elements called neurons. The neurons are organized in layers such that each layer receives input from its predecessor layer, performs some computation on the input, and sends output to a successor layer. A specific configuration of such layers is called a neural network, and the first layer in such a network is the input layer, while the last layer is the output layer. In between are one or more *hidden* layers. The depth in the term *deep neural networks* refers to the number of such hidden layers. Neural networks have proved to be universal approximators; given enough capacity, they can approximate any arbitrary function [82]. The output layer of a neural network can consist of a single neuron (predicting a continuous value for a regression model) or can be a cluster of *N* neurons with output probabilities corresponding to the class that each neuron represents (for an *N*-class classification model).

**Fig 5:**
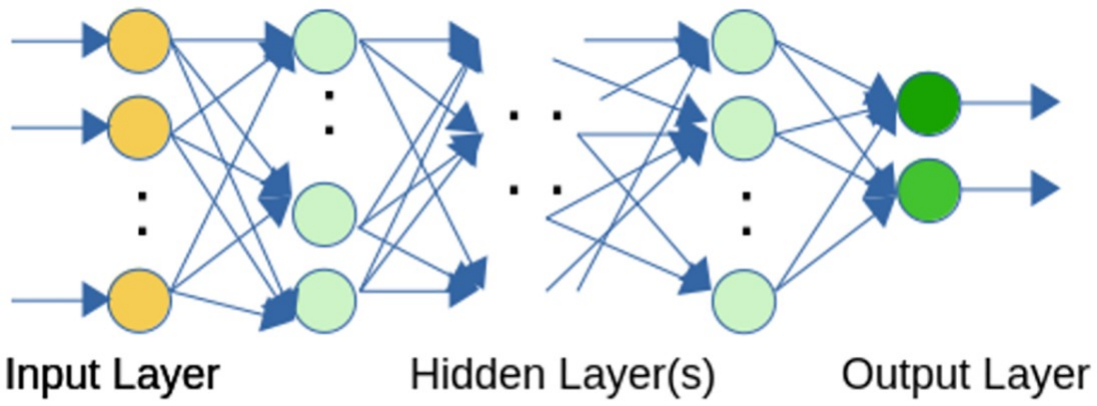
A feed-forward neural network.

A simple example of a neural network is shown in Fig 5. The input layer presents the initial data to the neural network; in the age prediction model, this data corresponds to the 11 features in Table 4. The input layer simply forwards the input data to the first hidden layer. All neurons from the first hidden layer to the output layer are organized into an input section and an output section. In the input section, an affine transformation shown in Eq 1 is applied to the data coming into the neuron:

$$\begin{array}{c} z=\sum_{i=1}^{n}x_{i}*w_{i}+b=w^{T}x+b \end{array}$$ (1)

Here, *x* is the input vector with *n* dimensions, *w* is the weight vector (also with *n* dimensions), and *b* is the bias term (the bias term has a corresponding bias neuron, which is not shown in Fig 5). The section of the neuron receives the affine combination in Eq 1 and sends through an activation function. The output of the activation function becomes the output of the neuron itself, and this output propagates to the downstream neurons. There are several activation functions defined in the literature, the more complex of which (sigmoid, tanh, etc.) serve to produce high-order, non-linear decision boundaries. In essence, the neural network learns by training itself on the training dataset where, during each pass of the training dataset, it adjusts the weight parameters. The output of training a neural network is the weight vector, which is subsequently used to predict an out-of-sample, previously unseen dataset.

**Fig 6:**
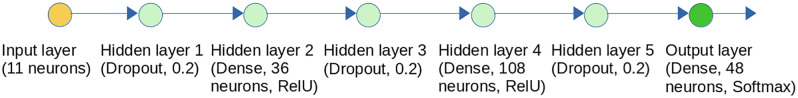
A simplified graph for the age prediction neural network.

We created a neural network to predict age as a regression model, experimenting empirically with various feedforward neural networks with a range of hidden layers (three, five, and seven), finally settling on a network consisting of five hidden layers as well as the network’s input and output layers. The neural network logical diagram is shown in Fig 6.

The input layer consists of 11 neurons corresponding to the predictors shown in Table 4. The first hidden layer is a dropout layer with a dropout rate of 0.2 (a *dropout* layer is used to combat overfitting in neural networks; the dropout rate indicates the fraction of neurons in that network that will not fire, or activate through an activation function). The second hidden layer consists of 36 neurons using the RelU activation function [83], and the output from this layer is sent to the third hidden layer, which is another dropout layer. The fourth layer consists of 108 neurons, also using the RelU activation function. This layer sends the results to the last dropout layer, which in turn sends the results to the output layer. The output layer consists of 48 neurons: if we consider each age as its own bin, then the problem can be abstracted as a multi-class classification problem. We felt that 48 bins cover the range of ages we are likely to encounter; for example, if young adults start to use cellular phones by age 13, the 48 bins will cover all ages between 13 and 61, inclusive. Any age above 61 would be fit the last bin. The Softmax activation function [77] is used in the last layer, this function produces a probability distribution consisting of 48 probabilities, summing to 1.0. The predicted age from the neural network will correspond to the neuron that produced the largest probability, using Eq 2:

$$\begin{array}{c} prediction=\underset{i\in \{0..|\vec{0}|-1\}}{\text{arg}\;\text{max}}\;\left[o_{i}=max\left(\vec{o}\right)\right]+min\left(\vec{age}\right) \end{array}$$ (2)

Here, $\vec{o}$ is the output from the neural network in the form a probability distribution ($|\vec{o}|=48$, the number of age bins), and $\vec{age}$ is the response variable. Predicted age is calculated by adding the minimum age in the dataset to the index of the neuron that produces the largest probability. We note that the calculation of the predicted age using Eq 2 may result in the leakage of age-related information from the holdout test set into the training dataset used since we need to know a-priori what the minimum age in the entire dataset is in order to train the model. Empirically, however, this particular information leak leads to better generalization since we do not have any observations in our dataset that have ages less than 13, or greater than 63, leading to inefficiencies as there is no training data to help the neural network model learn how to predict ages in these intervals.

In order to compute accuracy, we establish a range of ±*n* years, *n* ∈ {2, 3, 4, 5} around the predicted age. This interval represents the desirable range of the predicted age, i.e., ± 5 years represents a prediction interval of 10 years, while ± 2 years represents a prediction interval of 4 years. Intervals greater than ± 5 years were not considered as they represented a large age range; recall that the dataset was curated with the aim of inducing a model to predict customer demographics of pre-paid subscribers such that they could be converted to post-paid (monthly) subscribers. Accuracy is then defined as the fraction of predicted age that falls in the range of ±*n* years as shown in Eq 3:

$$\begin{array}{c} accuracy=\frac{p=\sum_{i=1}^{\left|D\right|}T\left(prediction_{i},n\right)}{\left|D\right|} \end{array}$$ (3)

where *p* is a count of correct predictions for a particular value of *n*, as shown in Eq 4, *prediction*_*i*_ is the predicted value from the neural network model for an observation, *D* is the dataset, and |*D*| is the size of the dataset.

$$\begin{array}{c} T\left(prediction,n\right)=\{\begin{array}{cc} 1: & age\in [(prediction-n),\;\ldots \;,(prediction+n)]\\ 0: & otherwise \end{array} \end{array}$$ (4)

where *age* is the real age corresponding to the observation.

Results from the neural network model are shown in Table 5 for the three distinct values of *n* and different number of hidden layers. As the table shows, the best accuracy was obtained for a 5-hidden layer model with a range of ±5 years. We note that similar accuracy was obtained when we split the sample by male/female and ran the neural network model separately for each. Gender differential, therefore, does not have a statistically significant effect on the predictive power of the age model.

**Table 5:** Neural network results on age prediction (accuracy).

	7 Hidden Layers	5 Hidden Layers	3 Hidden Layers
± 2 years	0.66	0.69	0.69
± 3 years	0.69	0.71	0.71
± 4 years	0.73	0.75	0.74
± 5 years	0.76	0.78	0.76

### 3.5 Gender prediction

We also explored the use of alternative classifiers in predicting gender to determine the algorithm that yields the best predictive accuracy based solely on network data. We found that a decision tree classifier resulted in an accuracy of 0.61, Random Forest yielded an accuracy of 0.65, and Gradient Boosting demonstrated an accuracy of 0.66.

**Fig 7:**
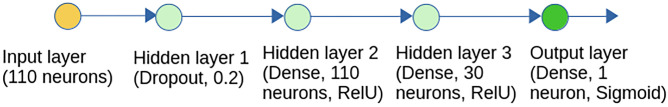
A simplified graph for the gender prediction neural network.

Gender prediction can be naturally cast as a binary classification problem; in developing a model to address the problem, we found that the available predictors in the dataset were not highly correlated with gender, as was the case for the age prediction model. Unlike in the age prediction model where we devised intervals to establish accuracy, however, the accuracy of gender prediction can be determined without intervals because of the binary nature of the response variable. We were also able to reduce bias by increasing the model complexity (number of predictors) while, at the same time, ensure that the resulting high variance did not impact the predictive power of the model. The final model has 111 predictors and yields a prediction accuracy of 0.67, which is generated by a neural network with the logical network diagram shown in Fig 7. Finally, Table 6 summarizes the accuracy of gender prediction for selected classification algorithms on the held-out test dataset. From Table 6(b) we can derive additional per class metrics; for instance the precision and recall for class M(ale) are 0.68 and 0.69, respectively, with an F1 score of 0.68. Similarly, for class F(emale), the precision and recall are 0.67 and 0.66, respectively, leading to an F1 score of 0.66.

**Table 6:** Gender prediction confusion matrices (F: Female, M: Male).

**(a)** Decision Tree, Accuracy: 0.61			
	Predicted		
Actual		F	M
	F	628	422
	M	430	678
**(b)** Neural network, Accuracy: 0.67			
	Predicted		
Actual		F	M
	F	693	364
	M	343	758
**(c)** Neural network, Accuracy: 0.65			
	Predicted		
Actual		F	M
	F	655	395
	M	349	759

This study was approved by Illinois Institute of Technology IRB, Protocol # IRB-2021–65, which has been filed with the publisher at the time of submission of the initial version of this study. The IRB categories this study as Exempt Category 4, Secondary Research for which consent is not required.

## 4 Policy-based efforts in the context of machine learning

The debate surrounding the use of ML techniques as a policy tool continues [84]. On the one hand, they generate predictions that can help policy makers make decisions with regard to crime and finance [85], and they can be used to address health related outcomes [86–88], depression especially, or target individuals with better educational opportunities based on their location and age. From the perspective of the firm, a user’s online search patterns can also be triangulated with age and gender to create a comprehensive user profile, which would have significant commercial value if the telecommunications firms or the app developing entity uses them for marketing purposes [89, 90].

However, ML techniques leave gaps in the analysis given a lack of clear understanding of behavioral motivations [84]. More problematically, even with relatively benign data, one may make predictions about age and gender with reliable accuracy using neural networks. (We do not ignore the possibility that, even when personally identifiable information is unavailable, back-end systems (e.g., when personal information is stolen during a data breach, can identify an individual by correlating the international mobile subscriber identity with the name of the person that owns it.) Essentially, any data scientist with similar machine learning tools and skills can apply a neural network to produce the same findings. Given the possibility that certain features of the model may be more crucial than others, accuracy may be more tenuous and attempts to challenge user privacy may fail without the complete set of features identified in this study. This could result in a flawed identification process with potentially similar outcomes to false-positive facial recognition technology [11–13, 91]. In light of the data available to telecommunications firms and the privacy-related risks for users, we recognize the need for a robust set of policy tools with the potential to reduce negative outcomes arising from predicting user demographics from anonymized datasets.

At the center of efforts to secure the individual’s online privacy rights is ambiguity about data ownership as well as the inability for users to understand precisely what information they are releasing to firms when signing up for app-based services. Abuse can occur if firms knowingly share stored information with third parties, or when malicious hacking results in the data being used to explicitly cause harm to users. Clearly, the telecommunications firm has ownership rights to data like those examined here. Yet, at the margins, there is ambiguity as firms—intentionally or otherwise—use their data in ways that transcend existing rules. For example, the Cambridge Analytica example [24] illustrates how violations of potential rules and user agreements occur when wireless phone users’ activities are monitored by network providers. Similarly, app stores such as the Google-Play store can become clearinghouses for personal information such as biometric ID, human behavior, and location, among others, when app owners aggregate permissions across all of their affiliated apps [92].

In the U.S., the public is only marginally responsive to these types of privacy-related concerns, reflecting a lack of policy activity around this issue [93]. Most people believe that their cell phone use is tracked by some form of private entity, whether it is an advertiser or a technology firm, and they are not confident that private firms would publicly acknowledge the misuse of users’ data or instances when users’ data have been compromised [94]. This is juxtaposed with the European Union’s GDPR, which was designed to provide users with a direct role in the management of their online personal information. GDPR and a number of policy prescriptions floated in the U.S. [95], including the California Consumer Privacy Act of 2018 (CCPA [96]), may attempt to minimize privacy-related transgressions online. However, despite the best intentions of these regulatory frameworks, they remain inefficient in the presence of ML techniques, which, given the availability of data to be aggregated across multiple platforms [97], create opportunities for a neural network model like ours.

### 4.1 The promise and limits of GDPR

In the remaining discussion, we focus on *user* privacy, ensuring PII of the user remains private, instead of *data* privacy, i.e., ensuring privacy during collection and dissemination of data. For data privacy, there are cryptographic techniques to allow computation on encrypted data [98, 99]. However, these techniques are not used broadly as they are still evolving and in many cases remain computationally expensive with differing expectations of accuracy [100, 101].

The attention that GDPR gives to privacy-related concerns, particularly those inherent in ML techniques, has led some to place high confidence in the regulation [102]. Declared in Article 22 of GDPR is the right of the individual to not be subject to “decisions based solely on automated processing, including profiling, which produces legal effects concerning him or her or similarly significantly affects him or her” [23] (please see https://gdpr-info.eu/art-22-gdpr/. The phrase “automated processing” is widely understood to include any machine learning model that reaches a decision without a human being involved in that decision directly). We detail these and other aspects of GDPR here, but it should be made clear at the outset that we are not optimistic about the effectiveness of GDPR. In the absence of periodic updates to the regulatory framework, the continued development of machine learning techniques effectively chisels away at GDPR’s foundation.

GDPR does address important standards in terms of privacy and the non-use of personal information. Recital 26 (https://gdpr-info.eu/recitals/no-26/) states that “[p]ersonal data which have undergone pseudonymization, which could be attributed to a natural person by the use of additional information should be considered to be information on an identifiable natural person.” However, Recital 26 also states that “account should be taken of all the means reasonably likely to be used to identify the natural person,” which could require GDPR to address the ability of ML-related techniques to identify individuals. GDPR also calls for the pseudonymization of data and minimization of the data collected are outlined in Article 25 (https://gdpr-info.eu/art-25-gdpr/), providing a clear standard for protecting personal information.

Under GDPR’s Article 7 (https://gdpr-info.eu/art-7-gdpr/), consent for others’ use of one’s personal data must be clearly and explicitly demonstrated. Further, consent for the use of one’s personal data may be withdrawn with ease by the affected individual at any time, a fact that must be clearly articulated to the individual before consent is originally given. The quality of services provided to the individual must be completely unrelated to whether or not consent is given for the use of one’s personal data. On the back end, Article 17 (https://gdpr-info.eu/issues/right-to-be-forgotten/ and https://gdpr-info.eu/art-17-gdpr/) establishes a crucial right, colloquially called the “right to be forgotten.” As stated in this article, the data subject may invoke the right for entities that holding his/her personal information to erase such information without undue delay, with few exceptions (see paragraph 3, Article 17, https://gdpr-info.eu/art-17-gdpr/). However, this right to erasure does not apply to personal data that have been anonymized (GDPR Recital 26, https://gdpr-info.eu/recitals/no-26/), meaning that the numerous characteristics may be left available for ML analysis after being scrubbed of the user’s name and other unique identifiers. Yet, even when personal data are anonymized, our efforts here have shown that age and gender can be predicted high accuracy, and other research has shown that it is possible for a large portion of the (U.S.) population with only three data points—age, gender, and zip code [103]. We consider the “right to be forgotten” sufficiently important to present additional commentary on this in Section 4.2.

GDPR Article 5 (https://gdpr-info.eu/art-5-gdpr/) establishes important conditions on personal data, these being that (a) the data is processed lawfully, fairly, and in a transparent manner; (b) the data has “purpose limitations”, i.e., it is collected for a specified, explicit, and legitimate purpose; (c) data collection is adequate, relevant, and limited to what is necessary (“data minimization”); (d) the data is accurate and kept up to date; (e) data that permits identification of subjects is retained for a limited time (“storage limitation”); and (f) data is processed in a manner that ensures integrity and confidentiality of the data through technical or organizational measures (i.e., encryption, and the need to restrict information to a set of individuals in an organization). Similarly, Recital 71 (https://gdpr-info.eu/recitals/no-71/) makes it clear that one can elect to not have automated processing of their personal data; “profiling” that includes demographic, professional, behaviors, and geographic characteristics, among others, of an individual. Profiling is accepted if an individual’s “performance of a contract between the data subject and a controller” is at stake. (Recall that in GDPR, a *controller* is a natural or legal person, or some authority charged with determining the purpose and means of the processing of personal data.) In other words, and despite the fact that “producers of the products, services, and applications [are] encouraged to take into account the right to data protection when developing and designing such products, services and applications… to fulfil their data protection obligations” (https://gdpr-info.eu/recitals/no-78/), automated profiling and decision-making is allowed if there is a legitimate service being provided to an individual that puts the service provider—a credit provider, for example [104]—in a position of risk. Similarly, under GDPR, employers retain the right to process the personal data of their employees (GDPR Recital 155, https://gdpr-info.eu/recitals/no-155/).

In terms of protections and safeguards regarding racial, religious, etc. information, Article 9 (https://gdpr-info.eu/art-9-gdpr/) establishes safeguards on processing of personal data that reveals racial or ethnic origin, political opinions, religious or philosophical beliefs, and trade union memberships. Furthermore, Article 9 prohibits the processing of genetic and biometric data for the purpose of identifying a European resident, as well as data concerning health or data concerning a natural person’s sex life or sexual orientation. Building on this, with regard to age-related differences, GDPR Recital 38 (https://gdpr-info.eu/recitals/no-38/) acknowledges that children must be assigned specific protections given their inability to understand or be aware of the risks related to how entities may use their personal data, namely marketing and creating personality profiles. There is, however, no attention given to seniors that could similarly misunderstand or be unaware of these risks.

Finally, we must also recognize that, in spite of the privacy-related constraints imposed by GDPR, Article 6 (https://gdpr-info.eu/art-6-gdpr/) establishes controls on lawful processing of data. Specifically, under GDPR, in addition to any explicit consenting—i.e. clear awareness by the individual with regard to his/her privacy-related rights—the processing of an individual’s personal information is also awarded to pursue compliance with a legal obligation, or for a task carried out in the public interest. All of the reasons for use of an individual’s data beyond those to which he or she has explicitly consented may be considered loopholes.

### 4.2 ML-specific limits regarding “right to be forgotten”

It is instructive to observe how Article 17’s “right to be forgotten” interplays with machine learning models, as there are dichotomous forces in play here. Machine learning models require data in order to search for patterns, and often, the data consists of PII of the user. It is perhaps reasonable to assume that once a model has been trained on a dataset, which may contain PII of some users, that deleting the training data preserves the privacy of the user under Article 17; however, this is not always the case. In machine learning, there is a class of attacks called *membership inference attacks* [105] that can be used to learn about the individual observations used to train the model. Under such an attack, the aim is to determine whether a particular observation was used as part of the model’s training dataset. If an “attacker” is successful in launching a membership inference attack, the privacy of users whose observations were used to train the model will have been violated. Therefore, it would appear that even deleting the training dataset after building the model is not a sufficient condition to preserve the privacy of the users whose information appears in the training dataset.

Shintre et al. [106] argue that Article 17 can be upheld in machine learning models by implementing influence functions [107], differential privacy [108], and using aggregate data instead of raw data for training [109]. Briefly, influence functions measure the change in a model’s accuracy when a training point is removed from the training set, and differential privacy attempts to capture precisely how much additional information of an individual is leaked by participating in a training dataset, which would not have leaked otherwise. Furthermore, Shintre et al. [106] suggest that when a user asserts Article 17, all models that were trained on the user’s information must be retrained after excising the observations corresponding to the user. We believe that the mitigations proposed by Shintre et al. [106] are important steps on understanding how machine learning models impact Article 17, but require further reflection to account of opposing forces, as discussed next.

Imposing Article 17 on machine learning models is not a zero-sum game. The right to be forgotten, when applied to machine learning models, requires that opposing forces be taken into consideration, and two particular forces have manifested themselves here: model accuracy and model interpretability. Model accuracy considers how well the model performs on observations that are not part of its training data; models are invariably induced to demonstrate minimal loss and, therefore, exhibit high accuracy. Model interpretability, an area of ongoing research [110, 111], refers to how facile the inner workings of a model can be in order to facilitate the understanding of how the model arrived at a decision. Techniques such as influence functions, differential privacy, and data aggregation have an adverse effect on both model accuracy and model interpretability; accuracy suffers in particular if the training dataset is not sufficiently large from the outset, or if it is not representative of the real world. For such datasets, implementing Article 17 by removing the training observations corresponding to an aggrieved user may thus reduce accuracy and impact interpretability.

Agrawal et al. [108] show that using differential privacy in machine learning penalizes the differential model accuracy by as much as 15% (absolute), a sizable drop in accuracy. Furthermore, if the removed observation consists of a minority class, excising it would effectively prohibit the model from learning patterns on that entire class—particularly problematic if that class is of potential interest. Data aggregation imposes its own penalty on accuracy, making the problem more acute when studies involve comparisons of multiple datasets, each of which is represented in aggregate form [112]. In the field of machine learning, it is generally acknowledged that some hypothesis sets such as decision tree and regression lead to more interpretable models. Decision tree allow rules to be extracted from the induced model, and the learned parameters of a regression model allow the data scientist to understand the contribution of the predictor to the model. However, to comply with Article 17, the techniques proposed by Shintre et al. [106] would adversely affect model interpretability. Naive application of standard machine learning techniques to aggregated data will be vulnerable to the ecological fallacy [113]. Furthermore, dropping the training observations of an aggrieved user under Article 17 may impact a decision tree model such that it becomes hard to justify a decision, especially as we discussed earlier, if the removed observation corresponds to the minority class. (In certain decision tree algorithms, the leaves of the tree encode the class information in proportion to the fraction of labels present in the leaf: the class assigned to the leaf consists of the class corresponding to the majority of observations. If an adverse decision is made based on a razor thin majority of class observations at a leaf, it may become hard to uphold the decision if it disproportionately affects a user).

## 5 Policy prescriptions

Advances in machine learning have chiseled away a number of GDPR articles designed to protect consumers from the imminent threat of privacy violations. While the early efforts (Shintre et al. [106]) to impose Article 17 on machine learning models to mitigate privacy are welcome, we believe that there is more work to be done here, both from a technical perspective and from the regulatory approach. From the technical perspective, as we argue in the last section, it is naive to assume that simply deleting the aggrieved user’s observations from training datasets will not impact model accuracy and model interpretability. Rather, innovative techniques must be formulated such that training models will require an exceedingly smaller number of observations to demonstrate high accuracy. On this point, Sucholutsky et al. [114] propose a “less than one-shot” learning task, where a model must learn *N* classes from *M* observations under the constraint that *M* < *N*. Furthermore, techniques to generate synthetic data using Generative Adversarial Networks (GANs, Goodfellow et al. [115]) appear promising. In a GAN, two neural networks compete against each other, the generative network generates synthetic observations after having learnt from real observations, and the adversarial network learns to determine whether the synthetically generated observations appear real enough. Both these networks play off against each other, thus getting better over time. We envision that the generative GAN network can learn enough entropy from real observations to generate synthetic ones that will pass the muster of the adversarial network. Working in tandem, they have the ability to produce synthetic data that does not suffer from being associated with a particular human subject. Such data can be used to train other models.

Furthermore, the prescription of Shintre et al. [106] on excising an aggrieved user’s observations from all models where such observations were part of a training set creates a high administrative burden. The controller must track the aggrieved user’s observations among the many models utilizing them during the training process. To re-train all affected models by removing pertinent observations when an aggrieved user seeks the protections offered by Article 17 is hardly practical. Instead, we believe that the technology itself should aid in Article 17 resolution, see Sucholutsky et al. [114] for a relevant example. As a final policy prescription for upholding Article 17 in the face of machine learning models, we believe that the controller should be guided by a group of machine learning specialists that are aware of not only the best practices in the field, but also emerging work epitomized by Sucholutsky et al. [114], and Goodfellow et al. [115]. As the technology advances from research into mainstream use, we expect an inflection point will eventually be reached where machine learning models no longer erodes the protections offered by Article 17.

We turn our attention next to Article 6, which states that the processing of personal data is a lawful activity as long as consent is given. But consent usually is requested at the point when the individual’s focus is on getting a service to work and not on privacy risks. Service users are expected to be less inclined to give consent when they are made fully aware of the implications of their decision for privacy. More generally, Chapter 2 of the GDPR, to which Article 6 belongs, spells out the principles underlying the processing of personal data. Article 8, in particular, states that the processing of a child’s personal data would be lawful as long as the child is at least 16 years of age. Teens and even youths in the early 20s, however, are highly susceptible to coercion through the means of exploitative advertising. We have demonstrated that neural networks models are fully capable of predicting age with a high degree of accuracy, and thus potentially exposing the younger populations to the risks of deception. If gender can in addition be identified as our analysis shows, a highly personalized ad can be deployed to lure the most vulnerable.

One way to address these loopholes is by modifying the choice architecture, which refers to the intervention that makes it more likely for an outcome in line with the user’s best interest to be achieved. Individuals often make sub-optimal decisions that are detrimental to their own well-being in moments when attention is drawn to other goals that are perceived to be more pressing. When deciding whether to accept a service agreement, individuals are prone to making snap judgments because of the pressure to address the more pressing matter as quickly as possible [116]. Users in particular are more likely to neglect privacy concerns, despite serious long-term consequences, as their focus at that moment is on getting access to a service. One explanation for the impulsive decision to give consent is the tendency to underestimate the probability of an event that is far from the forefront of the decisionmaker’s mind. Losses are greatly discounted when it is perceived to be only probable in the distant future, and users fail to exercise caution because immediate access provides an instant gratification [117]. We have shown, however, that advances in machine learning have heightened the probability of privacy losses.

Further, an array of interventions should be designed to ensure that the short-sighted self will take steps to protect themselves from a future invasion of privacy [118]. Reminding users the consequences of a rush to judgment can be achieved by providing an oversized label in bold typeface akin to “Cigarettes cause fatal lung disease.” Instead of requiring consent before user is allowed to proceed, an alternative is to provide an opt-out choice (that is, “check this box to keep your personal information private”). In contrast to current practices, such a choice architecture is designed to give users the opportunity to not give consent by checking the box. Opting out is a user’s directive to refuse the disclosure of nonpublic personal information to a nonaffiliated third party [119]. The policy instrument is thus a regulatory framework in which service providers are required to provide users with an opt-out notice. Failure to comply would then be subject to disciplinary measures which may include, for example, financial penalties or suspension of license.

An alternative to the legal approach that punishes non-compliance is an incentive mechanism that rewards service providers for cooperative behavior. The GDPR endorses the use of certification mechanisms if they allow users to easily identify providers that have complied with data protection requirements (see Recital 100, https://gdpr-info.eu/recitals/no-100/). Privacy Shield, for example, is a government certification program originally designed to provide a stamp of approval for American companies that agree to abide by GDPR standards before they can move personal data originating in the EU across borders. Broadening the scope of the program to include others that serve primarily the domestic market, as well as those engaged with trading partners outside of the EU, should benefit companies that seek to establish their pro-privacy credentials. (On July 16, 2020, the Court of Justice of the European Union issued a judgment declaring invalid the EU-U.S. Privacy Shield program, see https://www.privacyshield.gov/Program-Overview. This decision does not relieve signatories of their obligations under the program, however, as it appears that programs similar to the now defunct Privacy Shield will remain important for transcontinental commerce).

We now turn our attention to user privacy. Privacy is only secured when both the government and the private sector play an equal role and responsibility. While many express legitimate concerns about the government spying on them, others are completely unaware of the fact that corporations already have access to some of their most private information. We acknowledge that, in liberal democracies, it is much easier for citizens to change the government than to remove a CEO or to affect the bottom line of a corporation. We recognize that such democratic levers of shifting power are not available, or are only partially available, in countries with nationalized economies or authoritarian rules. However, even in countries where the rights of the individual are enshrined in constitutions and protected by the courts, one must wonder where government protections exist in the context of privacy-related matters?

The answer may lie in a regulatory environment that strikes the right balance between privacy and the greater good of society, and the government and industry can work together to craft legislation that protects individuals. For example, the TRACED Act, signed into law in November 2019 [120] was endorsed by both the private telecommunication sector and the Federal Communications Commission (FCC) to prohibit predatory practices (robocalls) that caused financial distress to millions of consumers but does not stifle innovations in the area of Internet telephony. As well, there are special circumstances in which the logic of collective action needs to prevail, expressed as exceptions to the safeguards mentioned in Article 9 (https://gdpr-info.eu/art-9-gdpr/). During a pandemic like COVID-19, for example, an obstacle to the widespread and effective implementation of contact tracing may be the public’s willingness to allow public health agencies and app developers to use their phone’s location to help identify and track individuals that have come into contact with infected individuals [121, 122]. An argument could be made for ML techniques to be used in this instance in order to help fill in gaps in contact tracers’ knowledge with regard to coronavirus transmission pathways. Indeed, we have demonstrated that neural networks models are capable of identifying women as well as the adolescents and the elderly, all groups of which have been shown to be the most vulnerable during a catastrophic event [123, 124].

## 6 Discussion

Our work demonstrates that advanced ML techniques can be applied to data collected on the Internet to de-anonymize protected biometric characteristics such as age and gender. This work should be viewed in the context of a larger discussion that is currently occurring as data becomes plentiful, forcing public institutions and private corporations to deal with the aftermath of powerful ML algorithms. EU’s GDPR, selected articles of which we discuss at length in Sections 4 and 5; California’s Consumer Privacy Act [96], the Illinois Biometric Information Privacy Act [125], and the recent EU Digital Services Act (DSA) proposal [126] are all designed in part to ensure that the user’s preferences and constraints regarding privacy are taken into account when corporations use the data generated by the user.

Privacy is ever more important because the Internet has increasingly evolved to a two-sided marketplace in which service providers and users agree to enter what is perceived to be a mutually-beneficial transaction. Privacy is the apparent casualty of the exchange that occurs when users demand free applications and services, unwittingly surrendering certain aspects of their privacy rights as Section 5 above noted. This leads to companies, sometimes unknowingly, exploiting user information that should be kept private from third parties that do not even need the information in order to operate. The plethora of offers received by US consumers for identity theft insurance is a testament to this problem. As much as 28% Americans admit to suffering from major identity theft problems, and a majority of Americans (57%) are not confident in the corporations safeguarding their private data [94]. Given the two-sided marketplace arrangement that has allowed corporations to build large-scale platforms that track user’s preferences and behaviours, it is not surprising that governments may attempt to replicate it under the guise of national security [30, 95].

Looking ahead, we call attention to a number of avenues for future research while highlighting the most pressing caveats. Our experience has shown that the use of neural networks for the greater good must address challenges to external validity. The possible omission of certain populations, i.e., those not contributing information to the network telemetry data set, would effectively exclude those individuals from receiving any benefits or hazards from the predictive algorithm. Some have avoided this by including other sources of data about the population under analysis [127, 128], but predictive error can still persist. The task, thus, is to minimize it as much as possible while accounting for variations across sub-populations. As well, the extent to which our results are generalizable should be addressed in a future study that considers other countries beyond Latin America. In terms of caveats, we acknowledge the no-free-lunch theorem [129]: no one method dominates all others over all possible data sets. We also acknolwedge the challenges arising from the temporal effects embedded in the telemetry data, e.g., a user may well check their account balance before booking an Uber ride and then view their Instagram or WhatsApp messages while en route, requiring a recurrent neural network (RNN) architecture [79] in light of the sequential nature of the data. In short, there is considerable room for better-performing algorithms than ours to arise, which would further strengthen the case made in this paper.

## 7 Conclusion

At the outset of this paper we asked a two-fold question about policy: how easily can ML techniques be applied to network telemetry data, and second, how, at the policy level, can we thwart efforts to extract private information? Our analysis demonstrates that we can use advanced ML techniques to de-anonymize protected characteristics of users. Our neural network model generates an estimate for gender with an accuracy rate of 67%, outperforming decision tree, random forest, and gradient boosting models by a significant margin; the model can predict subscriber’s age with an even higher accuracy rate of 78%. Investigating relevant articles of GDPR, we examined the role of regulations, and concluded with the following policy prescriptions. First, GANs can be used to produce synthetic data that cannot be associated with a particular human subject. Second, the controller (the entity in charge of the processing of personal data) should be guided by machine learning specialists who are aware of the best practices and work emerging in the field. Third, the choice architecture should be modified so that individuals will make better decisions. Fourth, incentive mechanisms should be designed and then implemented to update existing non-compliance protocols.

In closing, we demonstrated that advances in machine-learning algorithms have chiseled away GDPR articles designed to protect vulnerable groups from the imminent threat of privacy violations.We encourage policy makers, planners, and technologists to consider our policy prescriptions.

## Supporting information

S1 Dataset (ZIP)
